# Evaluation of Allplex™ GI-Parasite Assay—A Multiplex Real Time PCR for the Diagnosis of Intestinal Protozoa: A Multicentric Italian Study

**DOI:** 10.3390/tropicalmed10080234

**Published:** 2025-08-19

**Authors:** Ester Oliva, Libera Clemente, Nicola Menegotto, Stefania Varani, Antonella Bruno, Raffaele Gargiulo, Luciana Petrullo, Claudio Farina, Annibale Raglio

**Affiliations:** 1 UOC Microbiology and Virology, ASST Papa Giovanni XXIII, 24127 Bergamo, Italy; cfarina.microbg@gmail.com (C.F.); araglio@asst-pg23.it (A.R.); 2 Parasitology Study Committee, Italian Clinical Microbiologists Association (CoSP, AMCLI), 20159 Milano, Italy; libera.clemente@asugi.sanita.fvg.it (L.C.); nicola.menegotto@aulss2.veneto.it (N.M.); stefania.varani@unibo.it (S.V.); gargiulo.raffaele@aou.mo.it (R.G.); luciana.petrullo@ospedalideicolli.it (L.P.); 3 Division of Laboratory Medicine, University Health Authority Giuliano-Isontina, 34074 Trieste, Italy; 4 UOC Microbiology and Virology, ULSS2 Marca Trevigiana, 31100 Treviso, Italy; 5 Department of Medical and Surgical Sciences, University of Bologna, 40126 Bologna, Italy; 6 SC Microbiology and Virology, IRCCS San Matteo Hospital, 27100 Pavia, Italy; antonella.bruno2310@gmail.com; 7 SSD Clinical Microbiology, AUSL Modena, 41121 Modena, Italy; 8 UOS Parasitology, UOC Microbiology and Virology, AO dei Colli, 80131 Napoli, Italy

**Keywords:** Intestinal protozoa, multiplex PCR, *Giardia duodenalis*, *Entamoeba histolytica*, *Cryptosporidium* spp., *Dientamoeba fragilis*

## Abstract

Background: The microscopic examination of stool samples remains the reference method for the diagnosis of intestinal protozoal infections; however, this technique is time consuming and requires experienced and well-trained operators. Therefore, there is a growing interest in molecular diagnostic techniques, including commercial PCR assays. The aim of this multicentric study was to evaluate a commercial real-time PCR for the detection of intestinal protozoa in fecal samples. Methods: The samples were routinely examined using conventional techniques, such as macro- and microscopic examination after concentration, Giemsa or Trichromic stain, *Giardia duodenalis*, *Entamoeba histolytica*/*dispar* or *Cryptosporidium* spp. antigens research, and amoebae culture. The samples were frozen by the participating laboratories, retrospectively extracted and examined with one-step real-time PCR multiplex using the Allplex™ GI-Parasite Assay (Seegene Inc., Seoul, Korea). Results: A total of 368 samples were analyzed from 12 Italian laboratories. Compared to traditional techniques, the sensibility and specificity of the real-time PCR kit were as follows: 100% and 100% for *Entamoeba histolytica*, 100% and 99.2% for *Giardia duodenalis*, 97.2% and 100% for *Dientamoeba fragilis*, and 100% and 99.7% for *Cryptosporidium* spp., respectively. Conclusions: The Allplex™ GI-Parasite Assay exhibited excellent performance in the detection of the most common enteric protozoa.

## 1. Introduction

Intestinal parasitic infections are widespread in low- and middle-income countries, with an estimated 3.5 billion cases annually [1,2]. Enteric protozoan parasites are responsible for a broad spectrum of clinical manifestations, ranging from mild gastrointestinal symptoms to life-threatening watery or hemorrhagic diarrhea, and to extra-intestinal localizations [3].

While *Blastocystis hominis* is the most common protozoan detected in stool samples [4], giardiasis and dientamoebiasis are the major cause of disease in terms of frequency, and cryptosporidiosis and amoebiasis are, respectively, the third and fourth leading parasitic causes of death worldwide [2]. Regarding the prevalence of these protozoa in Italy, several studies have shown that B. hominis, G. duodenalis, and D. fragilis are the most frequent, often causing co-infections [5]. For example, Clemente at al., has shown that out of 575 enrolled people, 85 (14.8%) were positive for *D. fragilis* and 37.7% of those had co-infection with *B. hominis* [6].

However, these infections are often neglected and underreported.

The diagnosis of intestinal protozoan parasites typically relies on the microscopic detection of trophozoites, cysts, and/or oocysts in human fecal samples [7,8]; this is a sharp contrast to other microbiological fields, where innovative modern technology has replaced the more classical diagnostic methods in the last two decades [9]. Molecular techniques are more difficult to apply for the identification of enteric protozoa than other infectious agents, due to the thick wall of parasite (oo)cysts, making DNA extraction difficult, and due to the high density of PCR inhibitors in stool samples [3]. Despite being the reference method, the microscopic examination of stool for the diagnosis of protozoan intestinal infections exhibits several drawbacks, as it is labor-intensive, time consuming and requires a high level of skill for optimal examination, which remains a major challenge for many laboratories in the northern hemisphere due to the low number of positive samples received annually [10]. Moreover, protozoan parasites are difficult to identify, particularly when they are present in low numbers [3]. In addition to the rapid processing of the samples to prevent morphological alterations, iterative stool specimens collected over a few days are usually necessary to increase sensitivity [11]. The sensitivity and specificity of microscopic detection of protozoan parasites are in fact both regarded as scarce, the technique being limited by its poor sensitivity and inability to differentiate between closely related species. For example, it is impossible to differentiate microscopically between the cysts of the pathogenic and the non-pathogenic species of *Entamoeba histolytica* and *E. dispar* [12,13].

Diagnosis of *D. fragilis* relies on direct visualization of the trophozoites in stained fixed fecal smears by light microscopy, as demonstration of the characteristic nuclear structure cannot be achieved in unstained fecal specimens. *D. fragilis* may be difficult to distinguish from non-pathogenic protozoa [14]. Therefore, alternative diagnostic methods have been developed to overcome the limitations of conventional microscopic techniques. For example, for *G. duodenalis* and *Cryptosporidium* spp. identification, direct fluorescent antigen detection by trained microscopists has shown better analytical performances than conventional microscopy. Nevertheless, fluorescent microscopy is still time consuming and requires skilled microscopists and appropriate equipment [15]. Enzymatic immunoassays and immunochromatographic tests are also available, improving the identification of *E. histolytica* and the analytic turnaround time for *G. duodenalis* and *Cryptosporidium* spp. diagnostic. However, analytical performances of these tests vary according to the targeted parasites and manufacturers, with false negative and false positive results still requiring confirmatory tests [16].

Today, nucleic acid amplification tests might offer more sensitive alternatives and allow the distinction between the potentially invasive *E. histolytica* and the non-pathogenic *E. dispar* [16,17,18,19]

Over the last 10 to 15 years, many clinical microbiology laboratories have been provided with facilities to perform molecular diagnostics and automated approaches based on PCR are becoming increasingly available for detecting intestinal parasites, being less time consuming and demonstrating higher sensitivity and specificity than conventional methods [12,18,20].

The aim of this multicentric study was to evaluate retrospectively the Allplex™ GI-Parasite Assay (Seegene Inc., Seoul, Korea), a multiplex real-time PCR for the detection of intestinal protozoa from fecal samples and to compare the results to conventional methods, such as macro- and microscopic examination after concentration, Giemsa or Trichromic stain, *G. duodenalis*, *E. histolytica*/*dispar* or *Cryptosporidium* spp. antigens research, and amoebae culture. The DNA was extracted with the Microlab Nimbus IVD system, which automatically performed the nucleic acid processing and PCR setup.

The benefits of using this targeted panel for optimal parasitological diagnosis are discussed considering our results.

## 2. Materials and Methods

### 2.1. Study Design and Sample Collection

We designed a national, multicenter study with the participation of 12 Italian laboratories from northern (n = 10), central (n = 1), and southern (n = 1) Italy, including laboratories in Bergamo, Bologna, Treviso, Pavia, Lecco, Napoli, Legnano, Modena, Verona, Ancona, Pinerolo, and Brunico. The samples were collected during routine parasitological diagnostic procedures from patients suspected of enteric parasitic infection and were examined using traditional techniques according to WHO and CDC guidelines [9,21]. The samples were examined routinely using traditional techniques: macro- and microscopic examination after concentration, Giemsa or Trichrome stain, *Giardia duodenalis*, *Entamoeba histolytica*/*dispar* or *Cryptosporidium* spp. antigens, and amoebae culture.

**Figure 1:**
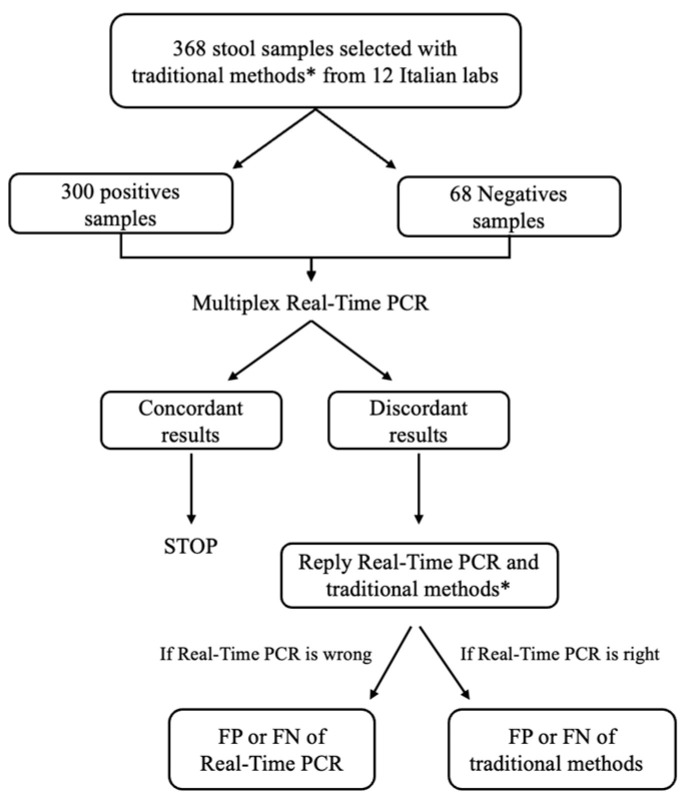
Schematic representation of the study flowchart. * Traditional methos: macro- and microscopic examination after concentration, Giemsa or Trichromic stain, *Giardia duodenalis*, *Entamoeba histolytica*/*dispar* or *Cryptosporidium* spp. antigens research, and amoebae culture.

A total of 368 samples were collected and stored at −20 or −80 °C in the different laboratories until they were sent to the Unit of Microbiology and Virology of Papa Giovanni XXIII Hospital (Bergamo, Italy) and tested by the Allplex™ GI-Parasite Assay (Seegene Inc., Seoul, Republic of Korea). In case of discrepancies in results, between real-time PCR analysis and traditional investigations, the samples were retested with both real-time PCR and traditional methods (Figure 1).

### 2.2. Real-Time PCR Assay

An amount of 50 to 100 mg of stool specimens was collected and suspended in 1 mL of stool lysis buffer (ASL buffer; Qiagen, Valencia, CA, USA). After pulse vortexing for 1 min and incubation at room temperature for 10 min, the tubes were centrifuged at full speed (14,000 rpm) for 2 min. The supernatant was used for nucleic acid extraction. Nucleic acids were extracted, using the Microlab Nimbus IVD system (Hamilton, Reno, NV, USA). The Microlab Nimbus IVD system automatically performed the nucleic acid processing and PCR setup.

DNA extracts were amplified with one-step real-time PCR multiplex (CFX96™ Real-time PCR, Bio-Rad, California, USA) with CFX Manager 1.6 software using the panel Allplex™ GI-Parasite Assay (Seegene Inc., Seoul, Republic of Korea). Fluorescence was detected at two temperatures (60 °C and 72 °C), and a positive test result was defined as a sharp exponential fluorescence curve that intersected the crossing threshold (Ct) at a value of less than 45 for individual targets. Positive and negative controls were included in each run. The identification panel included *Giardia duodenalis*, *Dientamoeba fragilis*, *Entamoeba histolytica*, *Blastocystis hominis*, *Cyclospora cayetanensis*, and *Cryptosporidium* spp. Results were interpreted using Seegene^®^ Viewer software (3.28.000 version). The PCR experiment was validated according to the manufacturer’s recommendations.

### 2.3. Statistical Assessment

The results were analyzed by descriptive statistics. Sensibility and specificity were evaluated for each pathogen detected by the Allplex™ GI-Parasite Assay. Microscopic examination, *G. duodenalis*, *E. histolytica*/*dispar* or *Cryptosporidium* spp. antigen detection, and amoebae culture were considered as the reference methods to calculate the sensitivity and specificity of the real-time PCR assay.

The multiplex real-time PCR results were considered true positive (TP) or true negative (TN) when in agreement with the traditional methods. The results were defined as false positive (FP) or false negative (FN) when discrepancies with the reference methods were observed after both the first and second evaluation (Figure 1).

To evaluate the performance between the traditional parasitological examinations carried out by the 12 laboratories and the real-time PCR under evaluation, the Kappa value was calculated. Results were interpreted according to the following Kappa values: (i) 0.01–0.20, slight agreement; (ii) 0.21–0.40, fair agreement; (iii) 0.41–0.60, moderate agreement; (iv) 0.61–0.80, substantial agreement; and (v) 0.81–1.00, perfect agreement.

### 2.4. Ethical Statement

The study protocol received ethical clearance by the Ethics Committee of Papa Giovanni XXIII Hospital, Bergamo, Italy (prot. Nr 172/19 date 19 September 2019).

## 3. Results

This study included 368 stool samples analyzed for the presence of enteric protozoa in 12 Italian laboratories. Specifically, among the 368 samples, microscopic investigation identified 78 negative samples [four samples were positive only for antigens (2 *G. duodenalis*, 1 *E. histolytica*, 1 *Cryptosporidium* spp.) and three fecal samples of patients who were serology positive for anti- *E. histolytica* antibodies, one positive only by *in-house* PCR for *G. duodenalis* used in routine analysis in one of the participant labs]. Microscopic investigations have also allowed us to identify: 18 non-pathogenic protozoa, 30 *B. hominis*, 52 mixed pathogenic and non-pathogenic protozoa, 87 *G. duodenalis*, 77 *D. fragilis*, 14 *Cryptosporidium* spp., and 12 *E. histolytica*/*dispar* (Table 1).

Sensibility and specificity for *C. cayetanensis* detection could not be evaluated because of the lack of positive samples.

Out of the 90 samples positive for *G. duodenalis* by traditional methods, 90/90 stool samples were also positive by the real-time PCR assay.

In addition, two samples were reported by microscopy as positive for *G. duodenalis*/*Cryptosporidium* spp. and *G. duodenalis*/*E. histolytica dispar*/*E. coli*, respectively, and were tested negative for *G. duodenalis*, *Cryptosporidium* spp. and *E. histolytica* by the Allplex™ GI-Parasite Assay; these samples were retested and confirmed to be negative.

The real-time PCR assays yielded two FP results in *G. duodenalis* detection. The two samples reported by traditional methods as positive, one for *Blastocystis* and one for *Cryptosporidium* spp., respectively, were confirmed negative for *G. duodenalis* both by repeating RT-PCR and traditional methods.

The molecular test under evaluation also found one new positive, confirmed as TP sample for *G. duodenalis*, which was reported from the labs, positive only for *B. hominis* and *E. coli* (Table 2).

Compared with the reference methods, the sensibility and specificity of the Allplex™ GI-Parasite Assay for *G. duodenalis* detection were 100% and 99.2%, respectively. In comparison the traditional methods for *G. duodenalis* presented sensitivity of 99% and specificity 99.2% (Table 2).

Regarding *D. fragilis*, out of 96 samples tested positive for this protozoan parasite by conventional methods 64 were confirmed as TP after comparison with real-time PCR results (Table 2). A total of 3 out of 79 samples that tested positive by conventional methods and negative by the molecular test were evaluated as FN; these samples were confirmed as positive by reviewing the previously stained slides. In addition, the real-time PCR test allowed the identification of 42 new positive samples that the conventional methods used by the laboratories participating in the study could not detect, confirmed as TP (Table 2). Following these results, the sensitivity and specificity for the real-time PCR were 97.2% and 100%, respectively (Table 2).

Of the 16 samples testing positive for *Cryptosporidium* spp. by conventional methods, 13 stool samples tested positive by Allplex™ GI-Parasite Assay, and 3 samples were evaluated as TN reviewing the slides, confirming the real-time PCR results.

One sample testing negative by conventional methods and real-time PCR positive for *Cryptosporidium* spp. was evaluated as FP; this sample was tested a second time by real-time PCR and was found to be negative. For the first test, the amplification cycles of the sample were too low (9 Ct), probably due to interference in the well.

One additional sample, testing negative by conventional methods and positive at the real-time PCR assay was confirmed as TP by microscopical examination and repetition of the sample (Table 2). As reported in Table 2, the sensitivity and specificity of the Allplex™ GI-Parasite Assay for *Cryptosporidium* spp. were 100% and 99.7%, respectively.

Regarding *E. histolytica*, the employment of real-time PCR assay allowed the differentiation between *E. histolytica* and *E. dispar* in three samples with negative microscopy, precisely two from patients with positive serology and one with positive antigen test, confirming them as TP. An additional sample tested positive only for *B. hominis* by microscopy was considered as TP for *E. histolytica*, in fact we discovered that the patient had positive serology for this parasite (Table 2).

As reported in Table 2, the Allplex™ GI-Parasite Assay exhibited 100% sensitivity and 100% specificity for *E. histolytica*.

By examining a large samples number, the present study indicates that the Allplex™ GI-Parasite Assay multiplex PCR offers optimal analytical performances, overlapping the routine parasitological investigation procedures performed by microscopists and traditional methods, and shown by the perfect agreement with the traditional methods as seen in Table 3 (Kappa values ranging between 0.96 and 1.0).

## 4. Discussion

Despite several limitations, conventional methods, including microscopic examination and antigen detection, still represent the gold standard for diagnosing gastrointestinal parasites [16].

*E. histolytica*, *G. duodenalis*, *Cryptosporidium* spp., and *D. fragilis* are the four most important and commonly occurring diarrhea causing parasitic protozoa and it is essential that a correct diagnosis is carried out for all four protozoa to reach successful treatment.

The study design, with the collection of stool samples in 12 distinct centers, allowed the inclusion of 368 positive and negatives samples for enteric protozoa. In this multicentric study, the Allplex™ GI-Parasite Assay for the detection of *Cryptosporidium* spp., *D. fragilis*, *E. histolytica*, and *G. duodenalis* was shown to be an optimal diagnostic tool for rapid, sensitive, and specific detection of these enteric protozoa. This assay combines several advantages such as it being an automated process and this technique could improve the routine diagnosis of protozoan infections by clinical laboratories as it is easier to implement compared to microscopy.

Considering what was published before [4,6,14,16], we observed that by employing the real-time PCR, the sensitivity of *D. fragilis* identification is higher compared to traditional methods. In this study, we show that traditional methods exhibit scarce specificity for the detection of *D. fragilis* in stool samples (Table 2), in fact of the 79 samples sent for the study that were positive for this protozoan, 15 were positive only for *B. hominis*.

Diagnosis of *D. fragilis* relies on the direct visualization of the trophozoites in stained fixed fecal smears by light microscopy, as demonstration of the characteristic nuclear structure cannot be achieved in unstained fecal specimens [14]. Traditional microscopic assessment encounters significant challenges to identifying *D. fragilis* trophozoites due to their rapid deterioration outside the intestinal lumen and the fragility of their binucleate structure [22]. Therefore, it may happen that *D. fragilis* has often been mistaken for *B. hominis* and vice versa, or both are present and only *B. hominis* is reported, so it is useful in the real-time PCR panel to have *B. hominis* to confirm microscopic examination.

Considering the data from the literature (Table 4), there is also increasing evidence that real-time PCR should be preferred over antigen detection for definitive identification of *E. histolytica*, in fact, we obtained a rapid and accurate identification, allowing the discrimination between the two pathogenic and non-pathogenic amoebas [13,16,20].

Regarding *G. duodenalis*, our work is in line with what has already been recently published in the literature, as can be seen in Table 4 [16,23].

The major limits of this study were the poor number of *Cryptosporidium* spp. and *E. histolytica* tested, in line with the low prevalence of these intestinal parasites in Italy [5]. Despite this, it was possible to evaluate the usefulness of real-time PCR.

Another potential confounding variable arises from the study’s reliance on conventional microscopic examination as the reference standard for diagnosing parasitic infections, despite the well-documented variability in sensibility that is dependent on the microscopist’s expertise.

The importance of this study is highlighted by the fact that we were able to define true positive and true negative samples; although the introduction of more sensitive molecular methods to verify the results, such as gene sequencing, would have been interesting.

Sensitive multiplex real-time PCR panels for molecular diagnosis of enteric protozoan parasites have been developed to overcome microscopy-based diagnostic limitations. However, the interpretation and clinical implications of positive real-time PCR results remain a challenge for the treating physician [24], in fact the increasing employment of molecular techniques could cause the loss of the experience of traditional parasitology, which is crucial to confirm indeterminate cases.

The examined panel Allplex™ GI-Parasite assay, could be employed in a diagnostic algorithm for a molecular screening of stool samples for enteric protozoa. Nevertheless, microscopy is necessary to minimize the chance of missing other parasitic pathogens, and to verify the results of the molecular methods, even when the prevalence of nonprotozoal parasites is low, which may be the cause in particular diagnostic settings or patient populations [25]. The use of real-time PCR will allow the detection of *Cryptosporidium* spp., *D. fragilis* and *E. histolytica* as not all laboratories correctly apply all the diagnostic techniques necessary for their identification. The increase in reported cases of *Cryptosporidium* spp. recently [26] could also be due to the spread of commercial real-time PCR tests in the labs routine.

## 5. Conclusions

In conclusion, the Allplex™ GI-Parasite Assay exhibited excellent performance in the detection of the most common enteric protozoa like *G. duodenalis*, *D. fragilis*, *E. histolytica* and *Cryptosporidium* spp.

To maintain high quality of care, tailor-made algorithms should identify specific high-risk patient populations who deserve in-depth diagnostic analysis and allow for the identification of rare parasitic diseases. For these patient populations, flexible and highly specialized diagnostic parasitological tools are still essential and should be maintained in designated referral centers [10].

About the implications for future research, our study demonstrates how the collaboration between laboratories is essential for the evaluation of commercial tests for fecal parasitological diagnostics as an important task for microbiologist associations, such as the Parasitology Committee of the Italian Clinical Microbiologists Association (CoSP-AMCLI). While PCR techniques are gaining increased attention in diagnostic laboratories for the development of reliable and cost-effective methods to identify fecal parasites, further studies are needed to standardize procedures for sample collection, storage, and DNA extraction, as these pivotal steps are essential for achieving consistent results.

## Figures and Tables

**Table 1:** First interpretation of the samples according to traditional methods. Footnote: * Parasite not identified by the Allplex™ GI-Parasite Assay (*E. coli*, *E. nana*, *I. bushlii*, *E. hartmanni*). ** Negative microscopy.

First Interpretation of the Samples According to Traditional Methods	N°
*G. duodenalis*	90
*G. duodenalis*/*Cryptosporidium* spp.	1
*D. fragilis*/*G. duodenalis*/*B. hominis*	1
*G. duodenalis*/*e. histolytica dispar*/*E. coli*	2
*G. duodenalis*/*B. hominis*	8
*G. duodenalis*/*E. coli*	1
*G. duodenalis*/*E. histolytica dispar*	1
*G. duodenalis*/*D. fragilis*	1
*G. duodenalis*/*E. nana*	1
*D. fragilis*	79
*D. fragilis*/*B. hominis*	16
*D. fragilis*/*E. nana*	1
*Cryptosporidium* spp.	15
*E. histolytica*/*dispar*	13
*E. histolytica dispar*/*E. nana*/*B. hominis*	3
*E. histolytica dispar*/*E. nana*	2
*E. histolytica dispar*/*E. coli*/*E. nana*	1
*E. histolytica dispar*/*E. hartmanni*	1
*B. hominis*	30
*B. hominis*/*E. coli*	6
*B. hominis*/*E. coli*/*E. nana*	3
*B. hominis*/*E. coli*/*E. nana*/*Chilomastix* spp.	1
*B. hominis*/*E. nana*	1
*Pentatrichomonas hominis*/*B. hominis*	1
Parasites not researched by the Allplex™ GI-Parasite Assay *	18
*E. histolytica* positive antibody **	3
No parassite found **	68
TOTAL	368

**Table 2:** Summary of results. ^(a)^ Calculated as follows: (number of true positives/[number of true positives + number of false negatives]) × 100. ^(b)^ Calculated as follows: (number of true negatives/[number of true negatives + number of false positives]) × 100.

	SENSITIVITY ^(a)^		SPECIFICITY ^(b)^	
	Traditional Methods	Allplex™ GI-Parasite Assay	Traditional Methods	Allplex™ GI-Parasite Assay
*Giardia duodenalis*	99% (104/105)	100% (105/105)	99.2% (261/263)	99.2% (261/263)
*Dientamoeba fragilis*	61.4% (67/109)	97.2% (106/109)	88% (228/259)	100% (259/259)
*Cryptosporidium* spp.	92.8% (13/14)	100% (14/14)	99.1% (351/354)	99.7% (353/354)
*Entamoeba histolytica*	87.5% (7/8)	100% (8/8)	94.4% (340/360)	100% (360/360)

**Table 3:** Results of the multiplex PCR assay compared to traditional methods. * (+/+): Positive by both traditional methods and Allplex™ GI-Parasite Assay (i.e., true positive sample, TP). (+/−): Positive by traditional methods and negative by commercial PCR assay (i.e., false negative sample, FN). (−/+): Negative by traditional methods/positive by commercial PCR assays (i.e., false positive sample, FP). (−/−): Negative by both traditional methods and Allplex™ GI-Parasite Assay (i.e., true negative sample, TN).

Parasites	(+/+) *	(+/−) *	(−/+) *	(−/−) *	K Test
*Giardia duodenalis*	105	0	2	261	0.98
*Dientamoeba fragilis*	106	3	0	259	0.98
*Cryptosporidium* spp.	14	0	1	353	0.96
*Entamoeba histolytica*	8	0	0	360	1

**Table 4:** Summary of recent results in the literature evaluating the sensitivity and specificity of commercially available kits (AusDiagnostic, G-DiaParaTrio^®^, RidaGene^®^) [16,23]. Se: sensitivity, Sp: specificity, n.a.: not applicable, n.d.: sensitivity and specificity not determined.

	AusDiagnostic (Nuclear Laser Medicine)		G-DiaParaTrio^®^ (Diagenode)		Rida^®^Gene (r-Biopharm)	
	Se	Sp	Se	Sp	Se	Sp
*G. duodenalis*	91%	98.9%	92.5%	100%	92.5%	99.3%
*D. fragilis*	68.4%	91.9%	n.a.	n.a.	25%	98.3%
*Cryptosporidium* spp.	78.9%	99.1%	100%	100%	92.3%	100%
*E. histolytica*	n.d.	n.d.	71.4%	100%	100%	100%

## Data Availability

All the data obtained from the analysis of the samples used in the study have been included in the article.
